# Surface Modification Techniques for Endothelial Cell Seeding in PDMS Microfluidic Devices

**DOI:** 10.3390/bios10110182

**Published:** 2020-11-19

**Authors:** Fahima Akther, Shazwani Binte Yakob, Nam-Trung Nguyen, Hang T. Ta

**Affiliations:** 1 Australian Institute for Bioengineering and Nanotechnology, the University of Queensland, Brisbane, QLD 4072, Australia; f.akther@uq.net.au; 2 Queensland Micro-and Nanotechnology Centre, Griffith University, Brisbane, QLD 4111, Australia; nam-trung.nguyen@griffith.edu.au; 3 School of Pharmacy, the University of Queensland, Brisbane, QLD 4102, Australia; shazwani.yakob@uq.net.au; 4 School of Environment and Science, Griffith University, Brisbane, QLD 4111, Australia

**Keywords:** PDMS, microfluidics, endothelial cells, surface treatment, hydrophobicity

## Abstract

Microfluidic lab-on-a-chip cell culture techniques have been gaining popularity by offering the possibility of reducing the amount of samples and reagents and greater control over cellular microenvironment. Polydimethylsiloxane (PDMS) is the commonly used polymer for microfluidic cell culture devices because of the cheap and easy fabrication techniques, non-toxicity, biocompatibility, high gas permeability, and optical transparency. However, the intrinsic hydrophobic nature of PDMS makes cell seeding challenging when applied on PDMS surface. The hydrophobicity of the PDMS surface also allows the non-specific absorption/adsorption of small molecules and biomolecules that might affect the cellular behaviour and functions. Hydrophilic modification of PDMS surface is indispensable for successful cell seeding. This review collates different techniques with their advantages and disadvantages that have been used to improve PDMS hydrophilicity to facilitate endothelial cells seeding in PDMS devices.

## 1. Introduction

Microfluidic technology, also known as lab-on-a-chip or micro total analysis system (μTAS), was applied in cell biology more than 20 years ago. Microfluidic techniques are powerful tools in cell culture because of its ability to create complex and controllable cellular microenvironment in microchannels [1]. This technology can provide a complex cell-based bioassay platform by integrating several steps such as fluid control, cell culture, cell capture, cell-cell and cell-matrix interaction, cell lysis, cell signalling, and detection of biochemicals in a single device [2]. Successful cell culture in microfluidic devices depend on the characteristics of the substrate materials. A broad range of polymers, such as polycarbonate [3], polystyrene [4], polymethyl-methacrylate [5,6], cyclic olefin polymers [7,8], and polydimethylsiloxane (PDMS) [9,10,11,12,13] have been used for fabricating microfluidic cell culture devices. Among them, PDMS has been gaining popularity because of the relatively low-cost and easy fabrication procedures as well as good mechanical stability [14].

PDMS is a silicon-based synthetic polymer, consisting of the repeating unit of Si-O molecules with two organic methyl groups attached to silicon. PDMS possess distinctive properties, including low elasticity, low thermal conductivity, high electrical resistance, chemical inertness, non-toxicity, non-flammability, and porosity [15]. Some intrinsic properties, such as biocompatibility, optical transparency and gas permeability can explain the acceptability of PDMS widely in a microfluidic devices for bioassay and real time imaging [13]. PDMS elastomer is transparent in the optical spectrum with wave lengths from 240 nm to 1100 nm [15]. The refraction index of PDMS is 1.4, making it compatible with various optical imaging methods [15]. Bright field imaging technique can precisely track, and image of small molecules or single cell in microfluidic device even at high frame rates [16]. On the other hand, the highly porous structure of PDMS allows for exchanging essential gasses (O_2_ and CO_2_) in a controlled manner for both short- and long-term cell cultures [13].

The main drawback of PDMS microfluidic devices in cell biology is the intrinsic high surface hydrophobicity. Due to its hydrophobic nature, PDMS surface possesses poor wettability with aqueous solvent [17]. However, most the biological experiments performed in microchannels need an aqueous solution or a mixture of organic and aqueous solutions [15,18,19]. Cellular attachment is strongly influenced by the physiochemical properties of PDMS, while the attachment might vary depending on the cell types [20]. Moreover, hydrophobicity might lead to absorption/adsorption of non-specific small molecules and biomolecules present in the cell media or secreted from the cells on the PDMS surface [21]. Cell signaling and behavior might be highly affected because of the depletion of biomolecules and secreted soluble factors [15]. To overcome this limitation, several surface modification methods are developed to increase the hydrophilicity by improving the wettability of the PDMS surface for facilitating cellular adhesion and proliferation in microfluidic devices.

This review summarizes the commonly used surface modification treatment, including plasma processing, coating with extracellular matrix (ECM) protein, chemical modification, modification with charged molecules, and improving surface roughness along with some combination techniques for facilitating endothelial cells (EC) seeding in PDMS devices. Confluent growth and proliferation of endothelial cells are pivotal to develop lab-on-a-chip platforms for studying vascular biology and diseases, inflammatory process, blood brain barrier, and diabetes. This review also highlights the common advantages and disadvantages of all techniques and provides an overview for selecting the appropriate modification techniques. Here, only a 10-part base elastomer and 1-part curing agent (10:1) ratio for PDMS device fabrication is considered to minimize the potential errors in comparing the ECs adhesion on the PDMS surface. Mainly, this ratio provides the optimum mechanical properties and biocompatibility for cell culture [15,22].

## 2. Fabrication of PDMS-Based Microfluidic Chips

Various methods have been developed and employed for the fabrication of PDMS microfluidic devices, such as soft lithography, inkjet printing [23], and direct writing [24]. Among these, soft lithography is a commonly used technique in PDMS chip fabrication for cell culture [13,15]. Soft lithography provides a simple, but a robust fabrication of microchannel with various patterns and high optical transparency [25].

**Figure 1:**
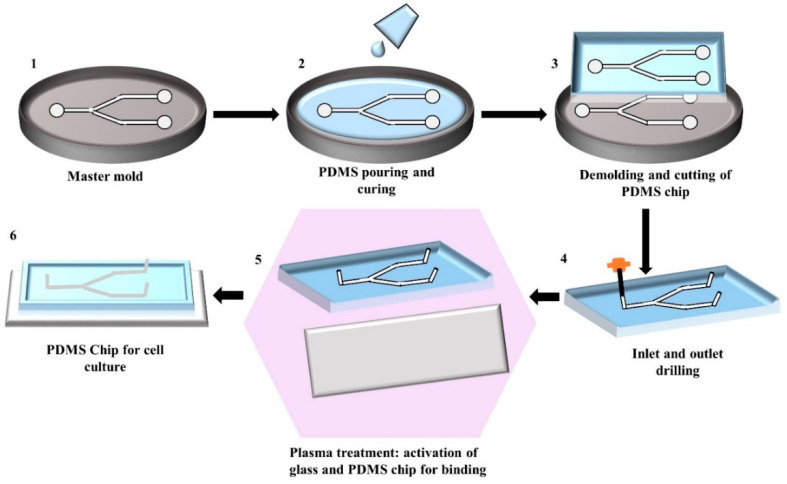
Illustration of the step-by-step fabrication process of a PDMS chip by replica moulding: (**1**) Generating silicon master mould using photolithography; (**2**) Pouring of the mixture of PDMS prepolymer and curing agent into the master mould and allowing it to solidify; (**3**) Peeling of the solidified PDMS from the master mould and cutting it into an appropriate shape; (**4**) Punching the inlet and outlet holes; (**5**) Activating the PDMS and glass surface by plasma treatment for facilitating the bonding; (**6**) Binding and curing of PDMS chip bonded on glass ready to use.

Soft lithography involves a group of patterning methods, such as imprinting, casting, and embossing with the elastomeric master mould or stamp [26]. PDMS exhibits a relatively low glass transition temperature and liquid at room temperature that makes it suitable to fabricate a replica from the master mould [13,17]. The two major steps in soft lithography are photolithography and replica moulding. Photolithography is used to generate the master mould. A photosensitive emulsion called photoresist is deposited on a silicon wafer and exposed to UV light through a photomask. To dissolve the unexposed regions, a developing reagent is used, and then finally releases the bas-relief structure of the master mould for PDMS fabrication [17]. A silicon master mould can be used several times for replica moulding. Replica moulding can be performed at ambient temperature. In general, liquid PDMS prepolymer is mixed with a curing agent at a ratio of 10:1 (base: curing agent). This ratio provides the optimum mechanical properties and biocompatibility for cell culture [15,22]. Mixing of PDMS prepolymer with the curing agent activates the polymer chains and transforms the liquid materials into solid elastomer. The time of PDMS curing normally depends on the temperature. PDMS can be cured within an hour at 75 °C while it can take 24 h at room temperature. After curing, PDMS device is peeled off from the master mould and small inlet and outlet holes are punched. At the final stage, the PDMS device is generally sealed to itself or another flat surface both reversibly, or irreversibly [27]. After bonding, the device is cured for 10 min at 75 °C and becomes ready to use. Figure 1 shows the step-by-step fabrication procedure of a PDMS device by replica moulding.

However, the common problem associated with the soft lithography technique is the deformation of patterns during demoulding [25]. This mould based technique requires an expensive photolithography technique to design the master mould that increases the production cost [28]. However, this technique does not require any clean-room environment during chip fabrication, the photolithographic master mould preparation needs to be done inside the clean-room environment [29]. Moreover, trained personnel and a well-equipped lab are required to perform this multi-step fabrication procedure.

## 3. Surface Treatment for Endothelial Cells (ECs) Culture in PDMS Microfluidic Devices

The hydrophobicity of PDMS is associated with the organic methyl groups present in the chemical structure of PDMS. Hydrophobicity of PDMS leads to poor wettability and limits the cell adhesion on the PDMS surface. Wettability is defined as the ability of the liquid to maintain contact with a solid surface and quantified by measuring the water contact angle (WCA). A surface with a WCA smaller than 90 °C is referred to as a hydrophilic surface, while WCA greater than this corresponds to a hydrophobic surface [30]. The WCA of PDMS is approximately 108 °C ± 7 °C [31], which makes the cell adhesion difficult on PDMS surface. Surface modification treatment is required to increase the hydrophilicity of the PDMS surface for optimal ECs adhesion. This section discusses about different surface modification techniques for ECs adhesion, and provides a summary of the recent studies (Table 1), with major pros and cons of different treatments.

### 3.1. Plasma Treatment

**Figure 2:**
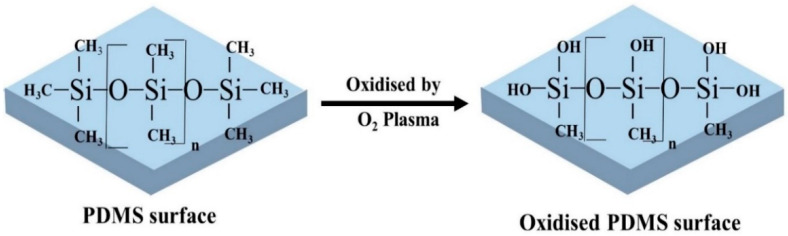
PDMS surface modification by oxygen plasma treatment. After oxidisation, the hydrophobic -CH_3_ groups are replaced by the hydrophilic silanol groups (SiOH) and improves wettability.

The most commonly used PDMS surface modification method is plasma treatment because the process is relatively simple and short [69,70,71]. Oxygen, nitrogen, argon, hydrogen bromide, and chlorine gasses are mainly used in plasma treatment [18]. These gases are dissociated and reacted with the PDMS surface to introduce chemical functional groups [31]. Among all, oxygen plasma treatment shows the most rapid increase of the hydrophilicity of PDMS surface by removing hydrocarbon groups and introducing polar silanol (SiOH) groups (Figure 2) via oxidization [18,72]. The enhanced hydrophilicity was evident with decreasing water contact angle (WCA) on PDMS surface by approximately 30° [32]. In a study done by Kühlbach et al. [33], human primary pulmonary arterial ECs were seeded into the PDMS device after plasma treatment. The cell confluency reached 100% just after 3 days and remained constant under continuous-flow (48 h) and pulsatile-flow conditions (72 h). The study suggested that cells secreted their own basement membrane that strengthened the cell adherence. Oxygen plasma treatment also helps to facilitate the adhesion of coating reagents such as ECM proteins, because of the increased hydrophilicity and wettability [34].

The main drawback of this technique is that the hydrophobic recovery of the PDMS surface occurs within an hour after exposure to air. Full recovery is obtained after 24 h air exposure, which limits the suitability for cell seeding [32,57,73,74]. Hence, instead of using a plasma-treated PDMS device for the cell culture directly, plasma treatment should be used to facilitate the adhesion of other coating materials for sustainable cellular attachment [35,75].

### 3.2. Coating with Extracellular Matrix (ECM) Proteins

In physiological conditions, vascular ECs are continuously exposed to the shear stress from the blood flow [76]. The fluidic stimulus could directly influence ECs alignment, morphology, proliferation, migration, gene expression, and functionalities [35,51], thus, cell seeding in microfluidic devices was often performed under “in-flow” conditions. The integration of microfluidic in cell culture provides an in vivo like platform to regulate the mechanobiological responses on chips [77]. Microfluidics allow control of the flow rate precisely through the channel, which introduces the required shear stress for cell alignment and proliferation. It is essential to achieve the stable anchoring of the cells with the coating substrates to withstand the applied stress condition and develop in vivo like endothelial lining inside the channel [35]. ECM proteins, such as collagen, fibronectin, and gelatin are usually used to coat PDMS to provide a natural moiety for the attachment and survival of cells [35]. ECM proteins showed self-assembly on PDMS surface by covalent bonding and facilitate the adhesion of ECs onto the PDMS surface by altering the surface roughness of PDMS [75,78]. Hong et al. used fibroblast-derived ECM to modify PDMS channel and seeded HUVECs (human umbilical vein endothelial cells) to study the HUVEC-ECM interaction under different shear stresses (0.5, 1, and 5 dyne/cm^2^) [56]. Shear stress was applied to the confluent cell layers for 2 h. To test the shear stress-induced stimulation on cell, VE (vascular endothelial)-cadherin (a biomarker of the adjoining cell-cell interaction) and vinculin (a biomarker of focal adhesion) orientation was observed. At a high shear stress (5 dyne/cm^2^), mature vinculin was found in long and thin lines while short line and dot formation was found in low shear stress (0.5 dyne/cm^2^). The similar pattern was observed for VE-cadherin, Furthermore, depolymerization of VE-cadherin was observed with an increase in shear stress that demonstrated the importance of shear stress in microfluidic endothelialisation. ECM proteins possess different cell adhesion moieties that can potentially improve cell attachment [52]. However, the addition and dissociation of the cells depend on the types of cells and matrix protein [79]. The main limitation associated with this technique is the dissociation of coating protein in a prolonged period [52,80]. This section discusses the commonly used ECM protein as coating materials for ECs seeding on a PDMS surface.

#### 3.2.1. Collagen

**Figure 3:**
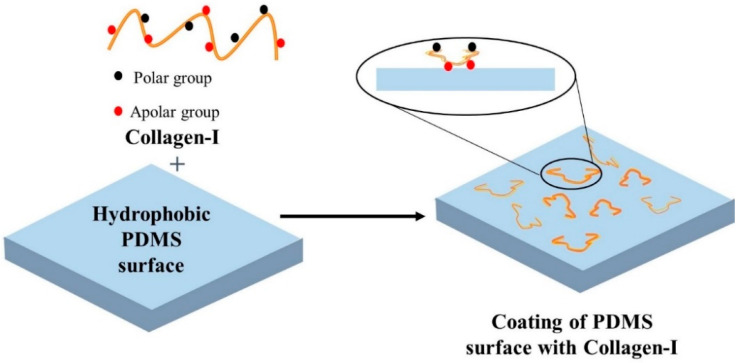
Schematic presentation of PDMS surface modification by collagen-I. The polar groups of collagen-I covalently bind with the PDMS surface and self-assemble to provide the platform for cell attachment.

Collagen is a major structural protein in the human body that can increase the hydrophilicity of PDMS [36]. Collagen type I is known to increase the hydrophilicity of PDMS to the greatest extent among ECM proteins, and is thus, the most commonly used for ECs seeding on PDMS surface [37]. The adsorption of collagen proteins on PDMS was also the highest among all ECM proteins [38]. Figure 3 shows a schematic diagram of PDMS surface treatment using collagen-I. Collagen, as a coating reagent, is relatively stable to shear stress. It is hypothesized that the triple helix structure of collagen interacts with the receptors present on the ECs membranes, allowing cell adhesion on a PDMS surface coated with collagen. A complex in vivo relevant vascular network was developed into a perfusable PDMS chip to study the large aid interaction of endothelial cells with drug, nutrition and waste under flow condition [39]. This study replicated the complex vessel architecture in three-dimensional (3D) microfluidic devices from small intestinal villi, pancreatic islets, and tumours of human and mice. To get the confluent layer of ECs through the complex network, the chip was treated with 0.05 mg/mL collagen solution and incubated at 37 °C for an hour before HUVECs perfusion. The cells were injected at 2.5 × 10^6^ cells/mL concentration and achieved confluency all over the vascular network within 5 days.

However, the long-term growth of ECs on collagen coating only is not optimal as it results in the formation of cell clusters and subsequent cell dissociation [52]. Thus, collagen might not be suitable for long-term experiments. Besides collagen type I, there is also collagen type IV. Collagen type IV decreases the water contact angle (WCA) of PDMS by approximately 10°, indicating it as a weak reagent to use for ECs seeding onto PDMS [81].

#### 3.2.2. Gelatin

Another ECM protein used for seeding ECs onto PDMS is gelatin. Gelatin is a collagen derivative and is a thermoresponsive hydrogel. Gelatin is non-toxic, biocompatible and biodegradable in nature. Gelatin is used to coat PDMS surface as it possess the cell adhesion motifs [44]. HUVECs shows strong adhesion to PDMS coated with gelatin under flow [45,46]. The cells were also stable when exposed to the shear stress [46]. Shi et al. [44] developed a biomimicking bi-layer vascular microfluidic model for antitumor drug testing. For this design, the first requirement was to achieve a confluents layer of ECs through the channels before seeding tumor spheroid laden Matrigel for establishing a co-culture tumor model. This study used porcine gelatin to improve the hydrophilicity of the PDMS surface. PDMS channels were coated with 0.2% gelatin solution and stored overnight before seeding bovine aortic ECs. ECs were infused through the gelatin coated chamber at 1 × 10^7^ cells/mL concentration and attached on the channel surface after 3 h of incubation. A confluent monolayer was established after 2 days and cells remained viable in longer period. In another study, 1% gelatin was used as a coating agents and confluency was achieved within a day after HUVECs seeding at the concentration of 2 × 10^5^ cells/mL [47] that indicates the varying concentration of coating material might influence the cell attachment.

Gelatin can maintain the activity of cells over an extended period, compared to other ECM proteins, such as collagen and fibronectin, and this could be considered as a major advantage [44]. However, when used alone as a coating reagent, gelatin in terms of ECs adhesion, is not as effective as other reagents such as collagen and polydopamine (PD). ECs, such as sheep carotid arterial ECs has poor adhesion on gelatin coated PDMS as compared to fibronectin [48]. Gelatin caused cell aggregation and increased the probability of cell dissociation from PDMS surface of some cell types [49] that might limit the use of gelatin as a coating material to some extent.

#### 3.2.3. Fibronectin

Fibronectin is used to seed ECs because of its specific domains that interact with cell membrane receptors, thus, allowing the adhesion of cells onto PDMS. Fibronectin has the highest rate of protein adsorption onto PDMS [38,48,53]. In a microfluidic network, fibronectin is better than other ECM proteins, such as gelatin and collagen for the adhesion of sheep carotid arterial ECs, primary porcine aortic ECs, HUVECs and valve ECs [47,48,79,82]. Esch et al. [51] used fibronectin-coated PDMS microchannels (square and semicircular) to culture human HUVECs in both static and dynamic condition, in order to investigate the role of vessel geometry and shear stress on HUVECs activity. Under the static condition, HUVECs were seeded on the open channels and allowed to attach for one hour before covering them with the cell medium. The study found that the confluent layer of cells was established within 2–3 days in both geometries. The adherent junctions were developed between neighboring cells. Interestingly, focal adhesion of the cells was observed on the flat upper wall of the square channels and on the bottom wall of the semicircular channel. However, weak immunostaining of vinculin reflected the abnormal focal adhesion under static condition. For the dynamic condition, a continuous medium flow rates of 0.5, 3 and 7.5 µL/min created shear stresses of approximately 5 dyne/cm^2^, 30 dyne/cm^2^ and 79 dyne/cm^2^. In both vessel geometries HUVECs, under high shear stresses (30 dyne/cm^2^ and 79 dyne/cm^2^), formed focal adhesion on all sides of the fibronectin coated channels and developed a confluent cell layer with the adherent junction. The focal adhesion and the confluent layer of the cells were confirmed by the strong immunostaining of vinculin and VE-cadherin respectively. On the other hand, at the lower shear stress (5 dyne/cm^2^), both geometries were not able to form cell adherent junctions and showed weak focal adhesion, indicating the higher impact of shear stress in cell adhesion rather than vessel geometry. The study suggested that shear stress above a certain threshold is essential for ECs migration and proliferation in microfluidic device. Fibronectin coated PDMS device with two parallel chambers was used to study ECs and vascular smooth muscle cells (VSMCs) interaction by developing co-culture under flow (54). The cells remained viable in long term culture and at a given shear stress of 1–1.5 Pa and strain of 5–8% that corresponded to the physiological arterial stress and strain, VSMCs perpendicularly aligned with the ECs that influenced to change the ECs morphology.

Studies suggested fibronectin only allows for the adhesion of EC on PDMS for a period of approximately 4 days without exposure to the shear stress [48,55]. This is further supported by another study that observed more than 95% detachment of bovine aortic EC from fibronectin coated PDMS in 2 to 3 weeks under the static condition [55] that could be a major disadvantage for long term cell culture. Young et al. [79] investigated cell attachment on fibronectin-coated and collagen-I coated microfluidic devices under shear stress. Different concentrations of proteins (100, 2000, 500 mg/mL) were used to coat the devices. This study used primary porcine aortic ECs and valve ECs. Valve ECs showed better spreading over fibronectin in all concentrations while aortic ECs showed good distribution on collagen-I. To check the anchoring strength of the cells with coating substrates, the cells were exposed to different shear stresses for a certain period. The cells were exposed to lower to higher stress (11, 110, 220 dyne/cm^2^) and each stress was applied on cells for 4 min to check the cell dissociation over a 12 min period. The study showed that if the shear stress was switched from low to high, cells abruptly dissociated from the surface for 30 sec and remained constant over time until the high shear stress was applied. That finding indicated the cells did not dissociate continuously over time and responded quickly to each shear stress level. Valve ECs showed better stability on the abrupt changes of shear stresses compared to the aortic ECs. Valve ECs adhered relatively well to both proteins, but showed slightly stronger anchoring stability to fibronectin under different shear stresses. This study suggested that the type of cells and coating substrates should consider with shear stress for mechanobiological studies of the cells. Another study showed that HeLa ECs could not reach confluency on fibronectin-coated PDMS [19]. This indicates that the usage of fibronectin as the sole reagent might not be feasible for all types of ECs and long-term cell culture.

#### 3.2.4. Other Biopolymers

Other biopolymers or proteins such as laminin and different types of anchor peptides were also used to modify the PDMS surface [56,83]. However, the effectiveness of laminin when used together with oxygen plasma was less effective than other ECM proteins [56]. For example, the WCA of laminin-coated PDMS surface was 47.6 ± 10.6° while the WCA of g collagen-coated PDMS surface was 31.0 ± 3.9° and 20.9 ± 5.1° [37]. A study investigated the anchoring properties of HUVECs on different ECM proteins such as fibronectin, laminin, and Matrigel [56]. After 6 h, the cells extensively spread over fibronectin and Matrigel while fewer attachment were noticed on laminin. After 24 h, high F-actin polymerization was also noticed on fibronectin and Matrigel coated device compared to laminin. Also, cells exhibited more in vivo like micro and nano structure when grew on fibronectin and Matrigel coated channel while clustered organization with short actin fibre was observed on laminin coated chamber. Therefore, laminin is not as commonly used as other ECM proteins.

### 3.3. Chemical Treatment

Chemical treatment of PDMS surface has been introduced because of ECM protein degradation, as well as instability under shear stress [57]. Chemically modified PDMS surface provides a strong and stable covalent linkage to cell adhesion moieties. This section discusses chemicals that are used for PDMS surface modification in cell culture.

#### 3.3.1. Coating with Silica-Titania

Silica-titania is a non-organic reagent. In terms of degradation, non-organic reagents have an advantage over biological reagents such as ECM proteins. Silica-titania can promote cell adherence onto PDMS as they form a thin SiO_2_ layer on the PDMS surface, thus, increasing the hydrophilicity and the chemical robustness of the PDMS surface and facilitates seeding of EC [66].

Typical silica-titania reagents are methyltriethoxysilane (MTES), tetraethylorthosilane (TEOS), and titanium isopropoxide (TISP). These reagents are used to cover the PDMS surface in different types of sol-gel combinations such as 60MTES/40TEOS, 70MTES/30TISP, and 80MTES/20TISP [67]. Such modifications could preserve the device geometry and optical transparency. However, cell attachment and proliferation on the modified chip could vary in different combinations of coating materials. Among these three sol-gel combinations, 80MTES/20TISP provided the most suitable environment for HUVEC adherence and growth, where it almost formed a monolayer on the channel surface. On the other hand, 70MTES/30TISP provides an intermediate environment for cell spreading and attachment, while 60MTES/40TEOS combination represents the most hostile environment with minimal spreading and attachment of cells. In this study, modified chips were reused several times for cell seeding that indicated good stability of such type of chemical coating.

#### 3.3.2. (3-Aminopropyl)triethoxysilane (APTES)

(3-Aminopropyl)triethoxysilane (APTES) is a silane coupling agent that is mainly used for immobilization of biomolecules. APTES increases the hydrophilicity of PDMS by forming amine functional groups on the PDMS surface. Amine functional groups can form hydrogen bonds with water and increase the hydrophilicity of PDMS surface. APTES as a single reagent decreases the WCA by approximately 70°, indicating an increase in hydrophilicity of PDMS surface [58,59].

In terms of adhesion, APTES coated PDMS allows the adhesion of cells as studies have shown that ECs are spindle-shaped when exposed to PDMS treated with APTES [59]. APTES modification showed a positive influence in ECs adhesion [59]. However, after the second day, the number of cells was the same for APTES treated PDMS and non-treated PDMS. As incubation time increases, cell proliferation further increased on the APTES treated PDMS surface [58].

When exposed to shear stress, PDMS coated with APTES showed good adhesion and stability for vascular ECs [60]. At the same time, the hazardous health consequences of APTES make it unsuitable for long-term investigations in microfluidic devices [52].

#### 3.3.3. Polydopamine (PDA)

**Figure 4:**
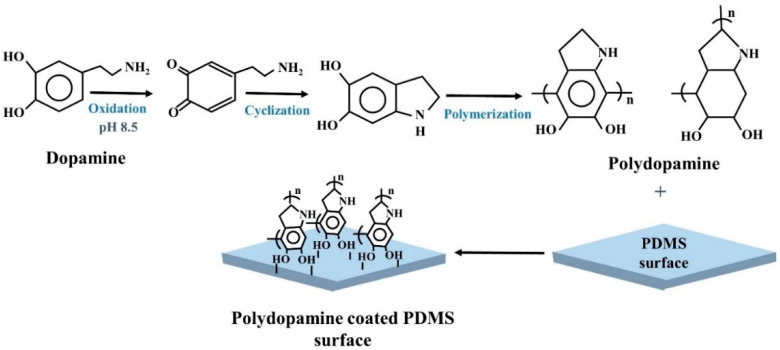
Schematic presentation of dopamine polymerization mechanism and PDMS surface modification by Polydopamine.

Polydopamine (PDA) has been increasingly utilized in PDMS surface modification. In alkaline condition, dopamine monomers undergo spontaneous polymerization and form PDA. PDA can bind to PDMS tightly through strong intermolecular interactions, such as covalent bonds (Figure 4). PDA modified PDMS surface can be used to control the adhesion of different cell types [61]. PDA is non-toxic to cells [49]. PDA improves surface hydrophilicity by reducing WCA and introduces different functional groups for bioconjugation [84]. PDA-treated PDMS surface showed 50% decrease in WCA as compared to untreated PDMS [62]. A flow based surface modification technique by using PDA was utilized to design a microfluidic cell culture device for organ-on-a-chip study [63]. Human cerebral microvascular ECs were seeded to create the blood brain barrier (BBB) on chip. ECs were able to show a tight junction protein named ZO-1 expression on day-7 that indicated the ability of ECs to form BBB on chip. Alhough, PDA coated PDMS device seldomly used for ECs culture but has been employed to enhance the cell attachment of bone marrow stromal cells. PDA-coated PDMS showed 40 fold increase of cell adhesion compare to the untreated PDMS by reducing the WCA of approximately 76° [52].

#### 3.3.4. Poly (Ethylene Glycol) (PEG)

Poly (ethylene glycol) (PEG) has been used to modify PDMS, serving as a medium between hydrophobic PDMS and external hydrophilic ECs, which facilitates EC adhesion. When PEG was used for the modification of the PDMS surface, the water contact angle decreased by approximately 57°, clearly indicating a significant increase in hydrophilicity of PDMS. However, the adhesion of HUVECs on the PEG treated PDMS surface was similar to the non-treated PDMS surface [64,65]. PEG was also used to encapsulate human induced pluripotent stem cells (HiPSC)-ECs. The cells were stable in the device for at least 2 weeks, making it suitable for moderately long-term cell culture [40]. It is better to encapsulate the cells in the PEG scaffold to improve cell adhesion instead of treating the PDMS surface with PEG.

### 3.4. Charged Molecules

Charged molecules such as poly-L-lysine have been used to increase the hydrophilicity of PDMS. Charged molecules possess either a net positive or negative charge, which forms an electrostatic interaction with ECs. Poly-L-lysine increases the hydrophilicity of PDMS to the smallest extent when used together with oxygen plasma, evidenced by the decrease in WCA by approximately 20° [37]. This study also suggested that ECM proteins, such as collagen, gelatin, and fibronectin had stronger cell adhesion properties compared to Poly-L-lysine.

### 3.5. Surface Roughness

Another method that aids the adhesion of ECs is modifying the surface roughness of PDMS elastomer. A study has done to observe the influence of PDMS surface roughness in the adhesion and elongation of rat aortic ECs [85]. Patterned PDMS films consisting of alternating grids of micro- and nano-rough topographies were moulded from titanium templates. Grid’s spacing was controlled by varying the dimension of micro- and nano- rough surface areas. Three types of patterned PDMS films were designed with the different grids spacing of alternating nano- and micro- rough topographies: 40 µm grid with micro-rough and 22 µm grid with nano-rough surface area; 35 µm grid with micro-rough and 48 µm grid with nano-rough surface area; and 45 µm grid with micro-rough and 80 µm grid with nano-rough surface area. Unpatterned PDMS films such as unmodified (smooth) PDMS film, entirely micro-rough, and entirely nano-rough PDMS films were used as control. Increase ECs adhesion was observed on all modified films while the highest adhesion, approximately 2-fold higher than the smooth PDMS, was observed on the entirely micro-rough film. Among the patterned films, PDMS with 45 µm micro-rough and 80 µm nano-rough alternating grids showed 58% increase of ECs adhesion than the smooth PDMS film. ECs showed enhance elongation on patterned films, compared to the non-patterned films. The highest elongation ratio of 1.9 was also observed on the film with 45 µm micro-rough and 80 µm nano-rough alternating grids that would make this combination a better choice for designing in vitro vascular graft. Although this type of modified elastomer has not been used in the fabrication of microfluidic devices yet, this could imply on preparing the chip.

### 3.6. Combination Treatment

**Figure 5:**
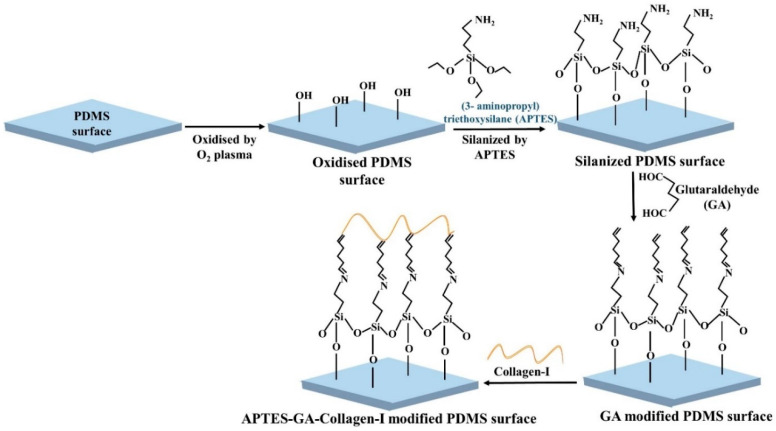
Schematic illustration of PDMS surface modification by APTES, cross-linker glutaraldehyde (GA), and collagen-I.

Combined treatments, such as ECM protein along with plasma treatment, chemical modification with ECM protein, and/or treating with different chemical reagents have attracted significant attention, and showed a higher success rate for cell attachment than individual treatment [19,35,37,49,52,64,66,86]. The modification of the PDMS surface with oxygen plasma along with the ECM protein coating increases the efficiency of cell seeding [37]. Zuchowska et al. [37] modified the PDMS surface with different proteins, such as ply-L-lysin, fibronectin, laminin, gelatin, and collagen-I alone, as well as the combination of oxygen plasma treatment and ECM protein and measured the hydrophilicity of the surface after each modification step. Compared to the ECM-coated PDMS surface alone, the combination treatment showed a higher reduction in WCA for all proteins. Gelatin and collagen-I with oxygen plasma treatment showed the highest reduction in the WCA of PDMS surface and for gelatin and collagen the amounted WCA was 21.3 ± 12.3°, and 20.9 ± 5.1°, respectively. Study suggested that covalent surface chemical modification of PDMS device with the combination of APTES, cross-linker glutaraldehyde (GA), and collagen-I (Figure 5) significantly increased the adhesion, spreading and proliferation of ECs, compared to the unmodified PDMS or the only collagen-I modified PDMS surface [87]. Similar modification was done by adding fibronectin instead of collagen-I [88]. PDA modified PDMS can drastically increase the adhesion of ECM scaffold and cell culture substrate [49,62,66]. A PDA functionalized PDMS device was constructed to maintain the long-term culture condition of vascular ECs and human lung fibroblasts co-culture in collagen-I [62]. PDA coated device provided a firm anchor for the hydrogel and maintained cell proliferation inside the gel for a month. An interconnected 3D networks was established by the self-assembly of ECs for 2 weeks. On the other hand, without PDA treatment, similar pattern of self-assembly of ECs was detected only for 2–3 days. In addition, hydrogel rapidly constricted and detached from the PDMS wall that resulted dense cellular aggregation. Besides using reagents to coat PDMS for cell seeding, physical methods were employed in combination with the reagents to enhance the adhesion of cells. One example is to rotate the PDMS device for a few hours constantly to facilitate cell attachment [66].

## 4. Conclusions and Perspective

PDMS is the most widely used polymer for the fabrication of microfluidic cell culture devices. The material catches the interest of biomedical researchers because of its chemical inertness and biocompatibility. Easy and low-cost fabrication methods also enhance the use of PDMS in lab-on-a-chip technology. The successful operation of PDMS microfluidic lab-on-a-chip mostly depends on the cell growth and proliferation in chip. However, the intrinsic hydrophobicity of PDMS can disrupt the optimal cellular adhesion inside the device. Cell adhesion and proliferation might depend on the ratio of the PDMS prepolymer and curing agents, and cell types.

Different types of surface modification treatments are performed to increase the hydrophilicity by improving the wettability of the PDMS surface for successful endothelial cells (ECs) attachment. Plasma treatment is the most commonly used modification method for PDMS, but the rapid hydrophobic recovery of the surface limits long-term cell attachment. Coating with different extracellular matrix (ECM) proteins deliberates an easy modification platform, while the increase of wettability varies among different proteins. Moreover, easy dissociation of coating protein under flow is commonly observed. Chemical treatment gives strong binding affinity to cells with PDMS surface. However, using a chemical could be harsh to cells and cytotoxicity must be checked carefully before use. Surface treated with charge molecules can bind with ECs by electrostatic interaction and can improve the adhesion propensity in some extent. Physical modification of PDMS surface, such as altering surface roughness can improve the cellular adhesion, but this method is only suitable for short-term cell culture.

As one treatment method has some advantages and disadvantages over other methods, it is important to combine different methods together to maximise the cell adhesion. However, one set of modification is not effective for all types of cell lines. Therefore, careful selection of methods and reagents are important for durable and cytocompatible PDMS modification for longer cell culture in dynamic condition. On the other hand, PDMS elastomer with different topographies modification could directly use in chip fabrication. This might help to omit the surface treatment complexity in micrometre scale and ease cell seeding inside the chips. Although, a PDMS chip for cell culture is still a new area, a continuous research for material and method selection, as well as designing new materials for achieving required PDMS properties is indispensable.

## Figures and Tables

**Table 1:** Summary of the extensively used PDMS surface modification treatment for improving cell adhesion.

Method	Hydrophilicity of PDMS	Type of Cell Used	Adhesion of Cells	Flow Conditions	Pros	Cons	References
Plasma Treatment	Increases as WCA decreases by approximately 30°	Human primary pulmonary arterial endothelial cells	100% confluency was achieved after 3 days on plasma treated PDMS surface	Confluency was equivalent in both static and flow condition	Relatively inexpensive Easy to perform. Time efficient.	The hydrophilicity of the oxygen plasma treated PDMS surface is temporary and gradual hydrophobic recovery is shown over time. It is not suitable for long term cell adhesion.	[32,33,34]
Collagen	Type I Collagen increases the hydrophilicity to the greatest extent among extracellular matrix (ECM)proteins	Human umbilical vein endothelial cells (HUVECs)	Both cell lines were able to attach and proliferate after initial seeding	Stable under static conditions for a few days	Good adsorption of collagen onto PDMS among ECM proteins Good modulation of ECs morphology Increases the hydrophilicity of PDMS to one of the greatest extents amongst reagents Exhibits good adhesion of ECs	Cell detachment occurs after a few days due to the formation of cell clusters Type IV Collagen is a poor reagent for seeding EC Might not be stable under high flow rates as ECs begin to detach at flow rates above 10 μL/min	[35,36,37,38,39,40,41,42,43]
		Endothelial cells derived from Human induced pluripotent stem cells (iPSC-ECs)		More cell activity than HUVEC under flow conditions of 10 μL/min			
		Human dermal microvascular endothelial cells	Confluent layer formed	Not specified			
		HUVECs	Good adhesion as confluency achieved after an hour	Cells were stable at flow rates of 5–10 μL/min			
Gelatin	Increases the hydrophilicity by increasing the surface roughness	Sheep Carotid Arterial endothelial cells	Poor adhesion of endothelial cells (ECs) as compared to other ECM proteins	Cells were adherent when exposed to the shear stress of 1 dyne/cm^2^	Able to maintain the activity of cells for the longest duration	Cell aggregation A high tendency for cells to dissociate from PDMS	[44,45,46,47,48,49,50]
		HUVECs,	Good adhesion				
Fibronectin	Hydrophilicity increases significantly	Sheep Carotid Arterial ECs	Good adhesion	Adhesion lasts for a few days without exposure to flow.	Second among the ECM proteins in seeding ECs The highest rate of reagent adsorption onto PDMS	Fibronectin is an ECM protein that can lead to cell dissociation	[19,38,48,51,52,53,54,55]
		HeLa ECs	Better than gelatin in terms of adhesion				
		Human aortic ECs	Unable to reach confluency				
		HUVECs	The same extent of adhesion as oxygen-fibronectin	Stable to flow rates at 7.5 mL/min			
		Bovine Aortic ECs	The same extent of adhesion as oxygen-fibronectin	95% detachment after 2 weeks under static flow			
Laminin	Increases but not as much as ECM protein.	HUVECs	Poor adhesion of ECs as compared to ECM protein.	Stable under flow at 5 dyne/cm^2^	Good adhesion	Spreading of cells over laminin-modified surface is slow. Might change the cell morphology.	[56]
APTES ((3-aminopropyl) triethoxysilane)	Increases as WCA decreases by approximately 70°	HUVECs	Cells proliferated with the increase in incubation time	Good stability and adhesion under shear stress (0.5 mm/s)	Chemical treatment is not prone to degradation Forms amine groups, which is suitable for HUVECs adhesion	Weaker increase in hydrophilicity as compared to ECM proteins	[57,58,59,60]
		Vascular ECs	Cell adhesion observed				
PDA (Polydopamine)	Increases as WCA decreases by 50%	Vascular ECs Human cerebral microvascular ECs	Improved adhesion and proliferation for both cell lines	Poorer response when exposed to flow compared to fibronectin	Significant increase in hydrophilicity Non-toxic to cells Long term stability for cell culture	Effect of PDA on cells is poorly understood Seldom used in ECs seeding	[49,52,61,62,63]
PEG (Poly (ethylene glycol))	Increases as WCA decreases by approximately 57°	HUVECs	Adhesion was similar to non-modified PDMS.	Poor cell adhesion under flow	Stable for long term culture when used to encapsulate cells	Poor adhesion when used as a coating reagent	[40,64,65]
		(iPSC-ECs)	When encapsulated with PEG, cells were stable for at least 2 weeks				
Silica-Titanium	Increases but less than ECM proteins	HUVECs	Good adhesion of cells	Not specified	Does not degrade easily as ECM proteins	Certain combinations of silica-titanium could present a hostile environment for cells	[66,67]
Oxygen Plasma + Fibronectin	Increases as WCA decreases by approximately 80°	HUAECs	The same extent of adhesion as fibronectin Confluency reached	Stable adhesion at physiological flow rate (0.5 mm/s)	Increases the hydrophilicity of PDMS to a huge extent	Cell dissociation in long term cell culture	[19,37]
PEG + RGDS (Arg–Gly–Asp–Ser) peptides	Increases	HUVECs	87% of cells coverage observed	Stable at low flow rates of 0.3 µL/min	Good adhesion of cells Cells increase with increasing RGDS density	The combination is not commonly used as ECM proteins	[64,68]
TEOS (tetraethylorthosilane) + Fibronectin	Increases	Primary Pulmonary Artery ECs	Adhesion of cells was achieved	Stable under low flow rates of 0.1 mL/h	Good adhesion of cells	The detachment of cells might occur at high flow rates	[66]

## Abbreviations

APTES	(3-Aminopropyl)triethoxysilane
EC	Endothelial cells
ECM	Extracellular matrix
HUVECs	Human umbilical vein endothelial cells
MTES	methyltriethoxysilane
PDA	Polydopamine
PDMS	Polydimethylsiloxane
PEG	Poly (ethylene glycol)
TEOS	Tetraethylorthosilane
TISP	Titanium isopropoxide
VSMCs	Vascular smooth muscle cells
WCA	water contact angle
